# The Fifth Year of the Century

**Published:** 1905-01

**Authors:** 


### Editorial.

### THE FIFTH YEAR OF THE CENTURY.

The average person, very naturally, grows somewhat sentimental at the opening of a new year in the calendar, but this seems unreasonable in view of the fact that every hour and every day should be held responsible for the development of human life.

The fifth year of the twentieth century! How many who now walk the world's pathways will be here when its last hour is rung out by the clanging of bells announcing its passage into oblivion and the birth of the twenty-first century of the Christian era? The human mind is incapable of realizing the full significance of that hour, the story which it will tell of the progress, the development of the human race.

It is possible to draw a picture of the future from the record of the past. The nineteenth century and its work will enable the philosophic mind to solve this in part, the destiny of this the twentieth century, which should be as much in advance as the former in all that makes for the progress of the world.

Professionally it is the part of wisdom not to indulge in, possibly, vain predictions. Indeed, the mind does not exist sufficiently clairvoyant to anticipate the progress hoped for and confidently expected in all branches of the healing art.

When the nineteenth century was young medical men were floundering in a wilderness of guesses as to the etiology of disease. The pathology of the period might be resolved in a series of negatives. "I don't know" was practically over every physician's doorway. That classical writer and profound medical teacher, Thomas Watson, in his celebrated "Lectures on the Principles and Practice of Physics in 1836," says, "During life it is often no easy thing to determine whether the parts of which the functions are disturbed preserve their integrity of structure or not, and even when the peccant organ is placed before our eyes after death, and the most careful scrutiny fails to discover in it any faultiness of texture, there may still be ground for suspecting that some material change, too subtile for detection by our senses, may have been wrought in its finer and more delicate organization."

Read the same author on the "Cause of Disease" and how poor it all seems in comparison to the knowledge obtained in the subsequent half-century upon the same subject. He says, in part, "The atmosphere may be a source of disease in consequence of its being loaded with impurities. Malaria, contagions of various kinds, and noxious gases in general may be considered as so many poisons." The word *may* indicates the sum total of the knowledge of the time in this direction. It can be said, with some degree of positiveness, that the genuine pathology of the human organism has all been worked up during the last half of the nineteenth century, and that all preceding this was but the shadow of the substance, that came rapidly in succeeding years.

That which is true of medicine is equally true of dentistry; indeed, it is true of all the specialties of the art of healing. While each and all of these are indebted to the past more than words can recount, yet the true renaissance did not appear until the professional mind became dissatisfied with the old pathology based upon mere guess-work.

This could not be said of practical dentistry. It grew upon a substantial foundation of fact, but these facts were limited. Its trade secrets led to no broad generalizations, and apparently were simply a means to gain the daily bread and butter and were without value in a professional sense. That came with the rise of medicine, and with it the stomatologist of the period is capable of developing the cause of disease in his domain of work on far more certain lines than was possible prior to 1850.

Men are living to-day, and actively engaged in professional work who are very familiar, from personal observation, with all the modern changes in medical knowledge and practice. These have come so rapidly that the student of the period regards them as having always been part of medical knowledge. They forget that the time from Leeuwenhoek to Cohn means an interval of innumerable investigations into the character and life-cycle of low forms of life. To the latter belongs the credit of increasing the interest in these problems, but from Koch (1881) the bacteriologist must date the beginning of bacteriology as a science.

Roswell Park, in his "History of Medicine," writing in 1899, says, "In civil hospitals, as well as in general and private practice, the mortality from these diseases (zymotic) was, until twenty-five years ago, simply frightful; while frequently, and over wide areas of territory, endemics and epidemics of puerperal fever would result in the death of almost every lying-in woman. In consequence of this terrible death-rate, surgeons were afraid to operate, and certain classes of operations, especially those on the abdomen and joints, were never performed except under the most exacting circumstances."

Thus prior to 1874 the germ theory of disease was practically an unknown factor in pathology, and from that period became an important subject in the curricula of the medical schools, as also did that of its sister science, histology.

Dentistry rapidly appropriated this valuable knowledge and dental pathology assumed an importance unknown in its past history, and the work of a large body of devoted laborers in this field of investigation have built a system through scientific development that has made dentistry a profession worthy the respect of this and future generations.

Combining these facts as a basis for prophetic knowledge, we can confidently look forward to the coming years as being full to overflowing of accurate information concerning the human organism in health and disease. In all directions this is being studied with an enthusiasm and self-devotion that insures an open way and a clearer understanding of the pathological problems that are, as yet, but a stumbling-block to the practitioner.

Hence we open this new year with hope. The year 1904 has been one of discouragement.

The golden era in dental education is not to be achieved by looking backward, but that has been the attitude of dental educators. They have gone back to the days of 1850, and have gloried in the results. The writer is not, however, disposed to pessimism. There are subtle forces at work in the world that no antagonisms can stifle. The laws of progress are eternal, and these will be obeyed and the efforts of the demagogue, the man of narrow ideas, the one who holds that "the ideas of the fathers are good enough for me," will be of no avail to force back the eternal roll of the ages to the new day and the higher conditions belonging to the more exalted ideas of a broader civilization.

It requires no prophet to foretell the destiny of the world, for past history demonstrates it. The refining processes that have been silently progressing for untold ages, from the coarser fibre of the flora and fauna of the carboniferous period and long antedating this, prove with mathematical exactness the progressive stages toward a higher standard of mentality in man and in everything in the physical world below him. Man's puny efforts to roll back this great ocean of progressive refinement will avail nothing, for all his efforts in this direction will but hasten the day of more perfect regeneration. Hence each recurring year is not a sad reminder of passing activities, but is a new source of satisfaction that we are one year nearer the goal, the final destiny of the human race; and not only this, but the entire world and all it contains; and as this law of progress applies to one world, so must it equally be part of the expansion of the universe.

